# Is adherence therapy an effective adjunct treatment for patients with schizophrenia spectrum disorders? A systematic review and meta-analysis

**DOI:** 10.1186/s12888-016-0801-1

**Published:** 2016-04-06

**Authors:** Richard Gray, Daniel Bressington, Ada Ivanecka, Sheila Hardy, Martin Jones, Michael Schulz, Suparpit von Bormann, Jacquie White, Kathryn Hoehn Anderson, Wai-Tong Chien

**Affiliations:** Health Services and Population Research Centre, Hamad Medical Corporation, Doha, Qatar; The University of South Australia, Adelaide, Australia; School of Nursing, The Hong Kong Polytechnic University, Hung Hom, Kowloon, Hong Kong; Independent Research Consultant, ADIGO, Bratislava, Slovakia; Northamptonshire Healthcare NHS Foundation Trust, Northamptonshire, UK; University College London, London, UK; University of Northampton, Northampton, UK; University of South Australia Department of Rural Health (DRH), University of South Australia, Whyalla Norrie, Australia; Diaconic University of Applied Sciences Bielefeld, Bielefeld, Germany; Institute of Nursing and Healthcare at the Medical Faculty of Halle-Wittenberg University, Halle-Wittenberg, Sachsen Germany; Research and International Affairs Boromarajonani College of Nursing Changwat Nonthaburi, Nonthaburi, Thailand; Learning Teaching and Quality, Faculty of Health and Social Care, University of Hull, Hull, UK; Center for Nursing Scholarship & Research, Georgia Southern University Nursing, Statesboro, Georgia USA

**Keywords:** Schizophrenia, Adherence therapy, Compliance, Adherence, Systematic review, Meta-analysis, Schizophrenia-spectrum disorders

## Abstract

**Background:**

Poor adherence to medication in schizophrenia spectrum disorders leads to inadequate symptom control. Adherence therapy (AT) is an intervention that seeks to reduce patients’ psychiatric symptoms by enhancing treatment adherence. We aimed to systematically review the trial evidence of the effectiveness of AT on improving clinical outcomes in these patients.

**Method:**

Systematic review and meta-analysis of published RCTs. We included studies testing AT as an adjunct intervention against treatment as usual or a comparator intervention in the general adult psychiatric population. The primary outcome of interest was improvement in psychiatric symptoms.

**Results:**

We included six studies testing AT in schizophrenia spectrum disorders published since 2006. A meta-analysis showed AT significantly reduced psychiatric symptoms compared to usual treatment over a follow-up period of less than 1 year. We found no significant effects of AT on patients’ adherence and adherence attitudes.

**Conclusions:**

AT is an effective adjunctive treatment for people with schizophrenia spectrum disorders.

**Prospero:**

CRD42015016779

**Electronic supplementary material:**

The online version of this article (doi:10.1186/s12888-016-0801-1) contains supplementary material, which is available to authorized users.

## Background

Maintenance treatment with antipsychotic medication is important for patients with schizophrenia [1]. Adherence to antipsychotic treatment is often poor; between 41 and 61 % of patients do not take medication as prescribed [2, 3]. Non-adherence can have serious consequences, including poor symptom control and an increased risk of relapse [4]. Effective interventions that have the potential to improve medication adherence may improve patients’ clinical outcomes.

Adherence therapy (AT) is a brief psychological intervention based on the principles of motivational interviewing (MI) and cognitive behavioural therapy (CBT). It was developed by Gray et al. [5] building on the work of Kemp et al. [6]. AT is a patient-centred approach normally delivered by trained clinicians over a series of 8 weekly sessions. Key therapy techniques include medication problem solving, exchanging information, exploring ambivalence, and challenging beliefs. Theoretically, these techniques amplify the personally relevant benefits of treatment, modify illness and treatment beliefs, and resolve ambivalence towards taking medication. The National Institute for Health and Care Excellence (NICE) [7] and the World Health Organization (WHO) [8] in their adherence guidelines review and advocate an approach to enhance adherence that concords well with AT. In particular, this should involve adapting the consultation style to the patients’ individual needs, establishing the most effective way of communicating with patients, encouraging patients to ask about their condition and treatment, and asking patients open-ended questions [7]. The NICE [9] and BAP (British Association for Psychopharmacology) [10] schizophrenia guidelines specifically recommend that AT is not used, creating a contradiction in the guideline recommendations.

The efficacy of AT on symptom outcomes has not been systematically studied. One previous systematic review by Hegedüs and Kozel [11] examined the effectiveness of AT on adherence. The review authors did not evaluate the effect of AT on symptoms and were not able to complete a meta-analysis because of missing data [12]. The aim of this systematic review was to determine the effectiveness of AT in addition to usual care on symptom severity and other outcomes in patients with schizophrenia spectrum disorders, when compared to treatment as usual alone or in combination with an active control. Symptom improvement is the focus of this review, primarily because a focus on improving adherence has previously been described as meaningless if patients’ clinical outcomes remain unimproved [13]. Other reasons for focusing on psychiatric symptoms include the widely-reported problems encountered when trying to accurately measure adherence and the fact that the majority of AT trials were powered to detect changes in symptoms, rather than treatment adherence [12]. Our secondary aim was to test the effects of AT on other patient outcomes, including adherence behaviour and attitudes.

## Methods

We adhered to the Preferred Reporting Items for Systematic Reviews and Meta-Analyses (PRISMA) guidelines for reporting the results of systematic reviews [14]. We registered the protocol for this review with the Prospero International Prospective Register of Systematic Reviews (http://www.crd.york.ac.uk/PROSPERO/display_record.asp?ID=CRD42015016779 number PROSPERO 2015: CRD42015016779).

### Search strategy

We conducted an electronic search of MEDLINE (1961–2015), Cumulative Index to Nursing and Allied Health (CINAHL with Full Text) (1904–2015), The Cochrane Library (1900–2015), EMBASE (1947–2015), PubMed and Scopus. The search strategy to identify relevant papers involved a MESH (or INDEXTERM) term ‘schizophrenia’ and keyword ‘adherence therapy’, combined using ‘and’ to identify papers reporting the effectiveness of ‘adherence therapy’ as described by Gray et al. [5] in patients with schizophrenia (see Additional file 1 for details). We also hand searched the reference lists from the included published articles to identify potentially relevant papers. We also contacted recent key authors to enquire about potential grey literature.

### Inclusion and exclusion criteria

#### Types of studies

We included all randomised controlled trials (RCTs) testing the effectiveness of adherence therapy (AT) [5] as an adjunct intervention with treatment as usual (TAU), compared with TAU or an active control. Studies were included if they were written in English and published between January 2006 (when AT was first described) and July 2015. We included studies with varying follow-up periods.

#### Types of participants

Participants with a formal diagnosis of schizophrenia spectrum disorders, including schizoaffective and schizophreniform disorders according to the criteria of Diagnostic and Statistical Manual, DSM-IV-TR [15] were included. Participants were aged 18 years or older. We included studies testing the effectiveness of adherence therapy within the general population of people with schizophrenia or related disorders. We excluded studies that focused on forensic patients because the additional legal restrictions and requirements for compulsory treatment may have influenced patients’ attitudes towards treatment. This would complicate direct comparisons of results within a general psychiatric setting. The study settings involved inpatients or outpatients treated in the community, who were receiving approved usual treatment for schizophrenia or other related disorder.

### Intervention and control conditions

We included RCTs published between January 2006 and July 2015 that tested the effectiveness of adherence therapy alone or as an adjunct therapy with TAU in people with schizophrenia spectrum disorders. Control conditions could either involve TAU, placebo or an active control treatment.

### Primary and secondary outcomes

The primary outcome in this review was psychiatric symptoms and secondary outcomes were medication adherence and adherence attitudes. Studies were included if they reported data for either the primary or the secondary outcomes, using validated quantitative questionnaires or other validated measures.

### Study selection and data extraction

The abstracts of studies identified from the search process were screened for eligibility by AI and DB independently. Papers with unclear eligibility were resolved by discussion. Full text articles were then obtained and read in detail independently by AI, DB and RG. The characteristics of studies viewed as being ineligible for inclusion were recorded in addition to the reasons for exclusion. All studies that reported the means and SDs of the areas of patient outcomes for the treatment and control groups were included in the meta-analyses. Where these figures were not available, attempts were made by DB and RG to obtain them from the researchers concerned. Data extracted from the studies included methodological information, descriptions of the experimental and control intervention, outcomes and their measures, statistical methods, length of follow-up, and description of the populations and setting(s). Data from studies was extracted independently by AI and DB and compared to eliminate errors.

### Risk of bias in individual studies and across studies

The studies included in this review were assessed for their quality using the Cochrane Collaborations’ risk of bias assessment tool [16].

We have potential conflicts of interest as we have been closely involved in conducting the included studies, therefore the risk of bias assessments were carried out by an external expert in systematic reviews, in addition to being assessed independently by members of the research team. The external reviewer’s scores and reasons for these were discussed at length in order to reach an objective consensus view. In case of queries, we contacted the trials’ authors to provide more information. We aimed to use the risk of bias assessments to contextualise the level of evidence for the review as a whole and highlight potential common biases across studies. The bias assessment was not used to determine the studies’ inclusion.

### Summary measures

In order that the results of the various studies could be compared and contrasted we calculated Hedges’ adjusted *g* standardised mean differences (SMD) and the 95 % confidence intervals (*CI*) for each of the clinical outcomes using Review Manager 5.3 software [17]. This was calculated as the difference between the means of the treatment and control condition at each post-test, divided by the pooled standard deviation.

### Synthesis of results

Due to the apparent degree of heterogeneity in terms of outcome measures used we conducted meta-analyses with SMDs using a random-effects model. The effect sizes for each study were pooled according to the model. We calculated *I*^*2*^ as an indication of the percentages of heterogeneity of pooled effect sizes, and tested the significance of heterogeneity using the *Q* statistic. The outcome assessment tools used in the studies measured three distinct areas of patient outcomes; symptoms, adherence attitudes and adherence behaviours. We therefore conducted a separate meta-analysis for each in line with recommendations from Higgins et al. [16]. We calculated overall effect sizes and 95 % *CI*s to estimate the intervention effects.

## Results

### Study selection

**Fig. 1:**
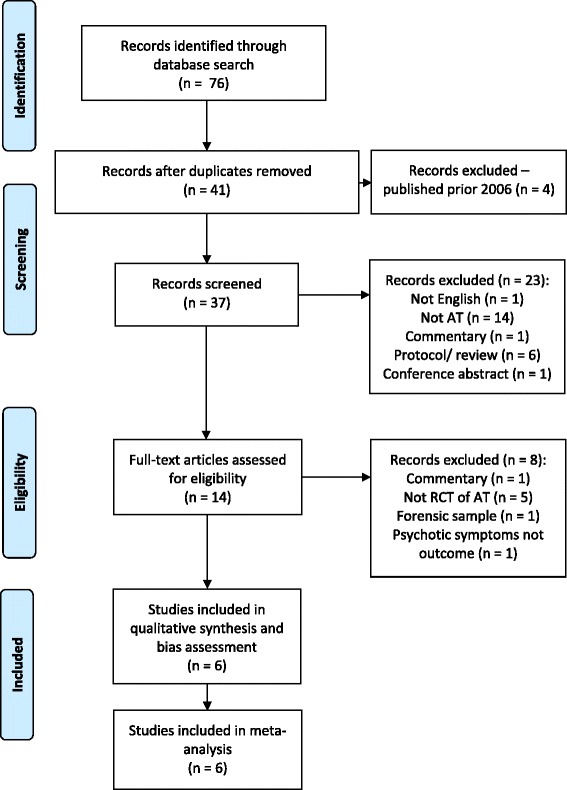
Prisma flow diagram of studies in the review

Figure 1 shows the results of the literature search within a PRISMA flow diagram.

Initially, 76 records were identified. After removing duplicates, 41 potential papers remained, of which four were excluded due to being published before the cut-off date (2006). Screening the remaining 37 papers’ abstracts and titles narrowed down the numbers of potential papers to 14. Of these, eight were excluded; the reasons for excluding papers were: commentary articles (*n* = 1), studies not reporting an RCT of adherence therapy (*n* = 5), one study involved patients within a forensic secure hospital, and one study did not report psychiatric symptoms as a study outcome. Six studies fulfilled the inclusion criteria for this review. One of the studies (Chien et al. [18]) reports outcomes at 6-month follow-up. We understand from the author that 12-month follow-up data will be reported in due course. We have only included the published data in this meta-analysis.

### Study characteristics

Table 1 shows the study characteristics and results of the studies included in the review.

**Table 1:** Characteristics of studies included in the review

Reference	Study location	Sample and setting	Interventions	Total participants *N* analysed at follow-up (intervention/control)	Baseline characteristics (intervention/control)	Number and duration of AT sessions	Follow up (attrition rate intervention/control)
Anderson et al. (2010) [19]	United States	Outpatients; diagnosis of schizophrenia or schizoaffective disorder aged >18	AT + TAU/TAU (day treatment, case management, employment placement, medication monitoring and individual counselling)	*N* = 23 (10/13)	Mean age 29 (13), range 21–57 in AT/31–62 years in TAU; 79 % male	Not reported	Within several days of completion (17 %/7 %)
Chien et al. (2015) [18]	Hong Kong	Outpatients; diagnosis of schizophrenia or other psychotic disorder within past 5 years; poor adherence (DAI score <11), recent non-adherence, aged 18–64	AT + TAU/TAU (routine treatment: psychiatric consultations at the two outpatient clinics, home visits by a community psychiatric nurse, brief education on psychiatric treatment and referrals to healthcare and welfare services)	*N* = 110 (54/56)	Mean age 29.21 (9.64) in AT/28.13 (8.96) in TAU; 51 % male in AT/53 % male in TAU	Mean n of AT sessions 6.9 (1.0)	6 months (3.5 %)
Gray et al. (2006) [5]	Amsterdam, Leipzig, London and Verona	Inpatients and community setting; diagnosis of schizophrenia; evidence of clinical instability in previous year;	AT + TAU/TAU + didactic health education	*N* = 371 (175/196)	Mean age 40.9 years (11.7) in AT/42.1 (11.4) years in TAU; 60 % male	Mean n of sessions 7 (1.96) in AT/7 (2.49) in TAU; mean duration 36 (12.1) min in AT/30 (9.9) min in TAU	52 weeks (12.7 %/5.4 %)
Maneesakorn et al. (2007) [21]	Chiang Mai, Thailand	Inpatients with community follow up; diagnosis of schizophrenia, aged >20	AT + TAU/TAU (standard care: medication treatment, occupational therapy, group counseling and recreational therapy)	*N* = 28 (14/14)	Mean age 38.7 (12.8) years in AT/43 (6.5) years in TAU; 81 % male in AT/61 % male in TAU	All (14) received 8 sessions of AT; mean duration 43.68 (6.24) min^a^	9 weeks (12.5 %/12.5 %)
Schulz et al. (2013) [20]	Germany: Bielefeld, Warstein, Lippstadt; Switzerland: Bern	Inpatients; aged >18, schizophrenic disorder and inpatient in participating ward	AT+ TAU/TAU (based on national guidelines; including medication, psychotherapy, occupational therapy and psycho-education)	*N* = 123 (72/51)	Mean age 35 (10) years; 60 % male in AT/56 % male in TAU	Mean number of sessions 7.24 (1.09; 5–9); mean duration of sessions 42 min (12.96; 17–92 min)	12 weeks
Von Bormann et al. (2015)	Thailand	Inpatients due to psychiatric exacerbation; aged >20, schizophrenia diagnosis	AT + TAU/TAU (medication, vocational and recreational therapy and outreach community psychiatric support)	*N* = 70 (38/32)	Mean age 38 (11) years in AT/40 (9) years in TAU; 71 % male in AT/78 % male in TAU	All received 8 sessions of AT; mean duration 41 (8.0) min	26 weeks
Total				*N* = 725 (363/362)			

Abbreviations: *N* number of participants, *AT* adherence therapy, *TAU* treatment as usual, *DAI* Hogan drug attitude inventor

^a^reported in Maneesakorn [39], a PhD thesis. Maneesakorn et al. [21] and Maneesakorn [39] reported the findings of the same study and are included as a single study in the review

### Participants

Participants’ baseline characteristics are shown in Table 1. The six included studies involved a total of 725 patients and were conducted internationally. Participants were patients with a diagnosis of schizophrenia or related psychosis such as schizoaffective disorder. Their mean age ranged between 23 and 41 years and in all studies the majority were male (range between 57 and 79 %). Anderson et al. [19] and Chien et al. [18] included outpatients with schizophrenia or other psychotic disorders, the remaining studies focused on inpatients with community follow up. Only one study explicitly focused on recruiting non-adherent patients (Chien et al. [18]). Gray et al. [5] reported that approximately 30 % of their sample was non-adherent. Patients were deemed mostly adherent in two trials [5] and [20].

### Study designs

Four of the six studies included in this review were described as being single-blind RCTs. Two (Anderson et al. [19] and Maneesakorn et al. [21]) were exploratory RCTs based on the definition from the MRC framework for the evaluation of complex interventions [22]. The follow-up period in the studies varied considerably, ranging between several days in Anderson et al. [19] and 1 year in Gray et al. [5], after completion of the intervention.

### Interventions

The experimental intervention in all six studies was adherence therapy. The control intervention in five studies was described as treatment as usual (TAU), which varied across studies, potentially due to their different settings. Only Gray et al. [5] offered didactic health education as control treatment in addition to TAU. Health education was provided in the same number and duration of sessions as was provided for the experimental treatment in order to control for the effect of the non-specific effects of time spent with health professionals [5]. Both AT and health education in this study were provided by the same therapists. Brief descriptions of TAU are shown in Table 1.

### Outcome measures

Table 2 shows the intervention outcomes for each included study. The numbers of participants for some areas of outcome are less than the total number in each trial due to missing data at follow-up.

**Table 2:** Intervention outcome

		Intervention group			Control group			Effect size
Study	Outcome measures	*n*	Baseline mean (SD)	Follow-up mean (SD)	*n*	Baseline mean (SD)	Follow-up mean (SD)	SMD (95 % CI)
Anderson et al. (2010) [19]	PETiT	10	40.10 (9.24)	37.30 (8.87)	13	40.10 (10.29)	41.61 (8.63)	−0.48 (−1.31, 0.36)
	PANSS	10	74.60 (13.79)	64.40 (30.54)	13	81.2 (17.66)	72.53 (19.20)	−0.32 (−1.15, 0.51)
Chien et al. (2015) [18]	PANSS	54	80.19 (11.10)	68.12 (14.81)	56	81.13 (12.01)	83.45 (14.13)	−1.05 (−1.45, −0.65)
	ITAQ	54	9.12 (6.14)	13.88 (6.80)	56	9.33 (3.31)	9.79 (6.21)	0.62 (0.24, 1.01)
	ARS	54	1.48 (0.98)	3.08 (1.24)	56	1.39 (1.01)	1.48 (1.01)	1.41 (0.99, 1.83)
Gray et al. (2006) [5]	SAI-C	173	5.04 (1.39)	5.22 (1.57)	189	4.73 (1.63)	5.03 (1.55)	0.12 (−0.08, 0.33)
	MAQ	172	2.98 (1.24)	3.20 (1.07)	194	2.97 (1.20)	3.33 (1.02)	−0.12 (−0.33, 0.08)
	BPRS	175	45.96 (13.23)	38.11 (11.33)	196	44.31 (12.79)	37.34 (9.79)	0.07 (−0.13, 0.28)
Maneesakorn et al. (2007) [21]	DAI-30	14	19.19 (6.96)	21.63 (5.91)	14	15.38 (9.82)	13.50 (7.58)	1.16 (0.35, 1.97)
	SWAM	14	116.81 (26.83)	126.50 (18.40)	14	115. 13 (20.79)	113.19 (19.12)	0.71 (−0.02, 1.40)
	PANSS	14	56.81 (10.86)	41.63 (10.33)	14	61.25 (15.58)	60.06 (13.94)	−1.46 (−2.31, −0.61)
Schulz et al. (2013) [20]	CDR	54	3.83 (6.80)	3.34 (5.36)	39	4.19 (5.79)	6.36 (10.56)	−0.38 (−0.79, 0.04)
	DAI-30	69	22.46 (6.83)	22.70 (6.59)	46	22.70 (6.69)	22.83 (5.89)	−0.02 (−0.39, 0.35)
	MARS	69	7.55 (2.07)	7.75 (2.01)	46	7.46 (1.73)	7.65 (1.87)	0.03 (−0.35, 0.40)
	PANSS	63	48.32 (13.83)	44.13 (10.67)	42	49.33 (14.74)	50.29 (13.67)	−0.51 (−0.91, −0.11)
von Bormann et al. (2015)	DAI-30	38	15.74 (8.85)	20.11 (4.79)	32	15.91 (7.69)	18.91 (7.24)	0.20 (−0.27, 0.67)
	PANSS	38	46.76 (16.06)	43.13 (13.92)	32	48.19 (16.05)	48.50 (15.42)	−0.36 (−0.84, 0.11)

Abbreviations: *ARS* adherence rating scale [30], *BPRS* brief psychiatric rating scale [24], *CDR* concentration to dose ratio, *DAI* Hogan drug attitude inventory [27], *ITAQ* insight and treatment attitude questionnaire [29], *MAQ* medication adherence questionnaire [31], *MARS* medication adherence rating scale [32], *PANSS* positive and negative syndrome scale [23], *PETiT* personal evaluation of transitions in treatment scale [28], *SAI-C* schedule for assessment of insight – compliance item [25]

Psychiatric symptoms were measured using the Positive and Negative Syndrome Scale (PANSS [23]) in five of the six studies. Only Gray et al. [5] assessed psychiatric symptoms using the Brief Psychiatric Rating Scale (BPRS [24]). Adherence attitudes were assessed in all six studies. One study [5] used the Schedule for Assessment of Insight – Compliance item (SAI-C [25]), two (Maneesakorn et al. [21] and von Bormann et al. [26]) used the Hogan Drug Attitude Inventory (DAI [27]); one (Anderson et al. [19]) used the Personal Evaluation of Transitions in Treatment scale (PETiT [28]) and one (Chien et al. [18]) used the Insight and Treatment Attitude Questionnaire (ITAQ [29]).

Adherence behaviour was assessed in three studies. Chien et al. [18] measured adherence using the Adherence Rating Scale (ARS [30]) that combines the ratings of two professionals. Gray et al. [5] used a self-rating scale Medication Adherence Questionnaire (MAQ [31]). Schulz et al. [20] evaluated patients’ adherence using an objective measure of medication concentration to dose ratio (CDR), in addition to patients’ self-rated adherence using the Medication Adherence Rating Scale (MARS [32]).

### Risk of bias across included studies

**Fig. 2:**
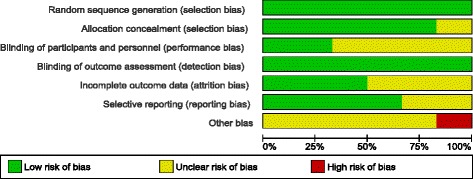
Overall risk of bias

**Fig. 3:**
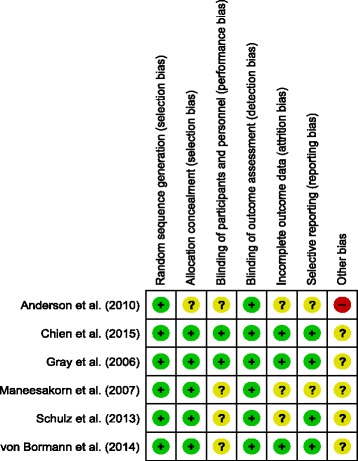
Risk of bias in each study included

Figures 2 and 3 detail the overall risk of bias and the bias assessment of individual studies.

We were able to obtain confirmation from the site coordinators, data analysts and co-investigators regarding bias risk issues through personal communication, but we decided that we would adopt a conservative and more objective approach towards all bias assessments by basing our scores on documentary evidence rather than personal report.

The study with the highest risk of bias included in this review was that conducted by Anderson et al. [19]; we decided that this study had an unknown risk of bias in most assessment areas except low risks of bias for “random sequence generation” and “blinding of outcome assessments”, but a high risk of potential for “other bias”. Issues that could indicate a high risk of “other bias” include an inadequately powered sample size, the follow-up being conducted immediately post-intervention and the high refusal/non response rate (80 %) of potential participants. Two studies (Gray et al. [5] and Chien et al. [18]) were judged to have the lowest risk of bias because the papers reported the selection process of participants and blinding issues in sufficient detail, in addition to accounting for any missing data and having enough information provided to determine a low risk of potential selective reporting.

All included studies presented a low risk of bias associated with random sequence generation because each study provided sufficient details about the methods used, or referred to an external randomisation service being used. We were therefore able to adequately determine that the process should have theoretically produced comparable groups. Allocation concealment was also generally well-described in all studies, except for Anderson et al. [19].

Due to the psychosocial nature of the AT intervention it was not feasible or possible for any of the participants or therapists involved in the included studies to be truly blinded to treatment allocation. This issue, in conjunction with a reliance on patient-reported outcome measures in the majority of studies is likely to present a risk of largely unavoidable participant performance bias and has resulted in us scoring four studies as an unclear risk of bias in this area ([19–21, 26]). The Gray et al. [5] and Chien et al. [18] studies were felt to have a “low” risk of performance bias when viewed within the context of the nature of the intervention tested. Gray et al. [5] reported that although participants would have been aware if they were receiving AT or health education, they were masked to which of the interventions was intended to be the control intervention. Chien et al. [18] used some more objective outcome measures in relation to levels of adherence and rates of re-hospitalizations. However, most studies, except Anderson et al. [19] provided enough details to ascertain that outcome assessors were blinded to treatment allocation.

The risk of reporting bias in two studies (Anderson et al. [19] and Maneesekorn et al. [21]) was rated as “unclear” because the treatment protocols were not published on an online trial registry and full details of participant attrition/exclusion were not reported in the papers.

All of the six studies were felt to have (at best) an unknown risk of “other bias” due to a range of reasons which included the use of treatment-as-usual as a control intervention (which does not account for the potential non-specific benefits of contact with therapists) and uncertainty that some studies established therapist fidelity to the manualised treatment. Unfortunately, high rates of refusal are common when conducting adherence studies. People who are non-adherent appear to be inherently less likely to agree to participate in research studies, resulting in potential selection bias and the recruitment of generally adherent participants [33].

### Results of individual studies

Table 2 outlines the results of each outcome area for individual studies. Of the six included studies, three found AT to significantly improve patients’ clinical outcomes compared to treatment as usual (TAU) (Chien et al. [18], Maneesakorn et al. [21] and Schulz et al. [20]) and the other three studies found no significant differences. Two RCTs (Chien et al. [18] and Maneesakorn et al. [21]) showed significant improvement of patients’ adherence attitudes in the AT group compared to TAU. Of the three studies reporting adherence behaviours as an outcome, only Chien et al. [18] found a significant effect of AT over TAU.

### Effects of interventions

We compared the effects of adherence therapy and control treatment on three outcomes of the individual studies: 1) psychiatric symptoms, 2) medication adherence and 3) adherence attitudes.

#### Adherence therapy vs. control treatment on psychiatric symptoms

**Fig. 4:**
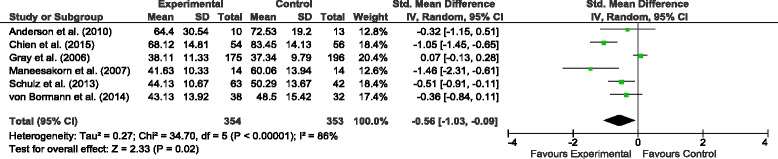
Comparison of the effects of the AT and TAU on psychiatric symptoms

All six studies reported the effects of AT and control treatment on patients’ psychiatric symptoms. Figure 4 shows the results of random-effects meta-analysis for the comparison of AT and control treatment on patients’ psychiatric symptoms, indicating a relatively high level of heterogeneity among the studies (*I*^*2*^ = 86 %; *n* = 6; 707 participants). Five studies found positive effects of AT over control treatment; and three of them were statistically significant (Chien et al. [18], Maneesakorn et al. [21] and Schulz et al. [20]). The meta-analysis of the pooled data showed a significant impact of AT on patients’ psychiatric symptoms with a SMD of −0.56 (95 % *CI −*1.03, −0.09; 707 participants) and effect size *Z* = 2.33 at *p* = 0.02.

#### Adherence therapy vs. control treatment on adherence attitudes

**Fig. 5:**
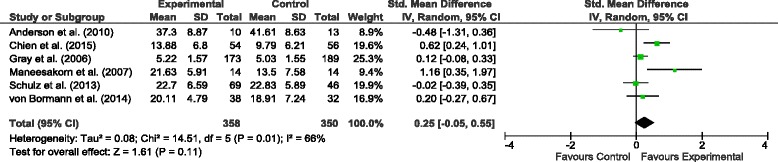
Comparison of the effects of the AT and TAU on adherence attitudes

All six studies reported a change in patients’ adherence attitudes. Figure 5 shows the results of random-effects meta-analysis comparing the effects of AT and control treatment on patients’ adherence attitudes. The overall effect (*Z* = 1.61) was not significant but favourable for AT, with the SMD 0.25 (95 % *CI −*0.05; 0.55). Between-study heterogeneity in adherence attitudes was considerable (*I*^*2*^ = 66 %; *n* = 6; 708 participants).

#### Adherence therapy vs. control treatment on adherence behaviour

**Fig. 6:**
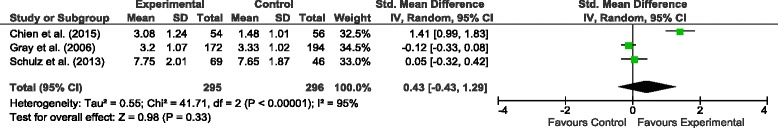
Comparison of the effects of the AT and TAU on adherence behaviours

Figure 6 shows the results of random-effects meta-analysis comparing AT and control intervention in patients’ adherence behaviours, which were reported in three of the studies (Chien et al. [18], Gray et al. [5] and Schulz et al. [20]). Between-study heterogeneity was high with *I*^*2*^ = 95 % (*n* = 3; 591 participants). The overall effect (*Z* = 0.98) suggests a favourable but non-significant trend for AT.

## Discussion

The aim of this systematic review was to evaluate the effectiveness of adherence therapy (AT) [5] on the psychiatric symptoms of patients with schizophrenia and related disorders. We identified six randomised controlled trials that mainly compared the effects of AT with TAU on patients’ psychiatric symptoms, medication adherence and adherence attitudes. We found that AT had significantly more positive effects on patients’ symptoms than TAU, but not on adherence behaviours and attitudes.

This is the first systematic review and meta-analysis evaluating the effects of adherence therapy on symptom outcomes in schizophrenia. The benefits of AT on patient outcomes are consistent with studies not included in this review, reporting that AT could reduce relapse rates in early psychosis [34] and/or improve psychiatric symptoms and adherence in forensic patients diagnosed with schizophrenia [35].

This review provides novel and important evidence that AT can improve psychiatric symptoms when compared to usual treatment. Our observation is not consistent with the current NICE [9] and BAP [10] guidance, which has concluded that AT should not be offered as a specific intervention for people with schizophrenia. Our meta-analysis of six RCTs demonstrated that AT could improve patients’ psychiatric symptoms, although the improvement in symptoms was less than the recommended clinically significant reduction of 20 % [36]. While the recommended reduction of 20 % was based on drug trials [36], we reviewed the effectiveness of a psychological therapy (AT) administered as an adjunct intervention. Its aim was to maximise the effects of usual treatment, rather than to act as a stand-alone intervention. In addition, in most of the included studies (except for Chien et al. [18]) the patients were notably less unwell than those included in many drug trials as they were only mild-moderately ill at the start of the AT interventions. The inclusion of mild-moderately ill participants in AT studies clearly leaves less room for potential improvement of symptoms. However, it is possible that a further reduction in symptoms of <20 % would be clinically meaningful and have a positive effect on patients’ outcomes.

The only trial where control treatment included an active contact with a therapist was Gray et al. [5] who provided didactic health education in combination with TAU. This was provided by the same therapists, potentially creating a significant risk of cross-contamination of therapeutic technique and effects. On the other hand, potential risk of bias by additional therapeutic contact was introduced in the other five studies where the control treatment involved only TAU. The overall risk of bias in the included studies was judged as mostly low or unclear. Consequently, even though there might be plausible bias that would influence the outcomes, this was not considered at a level that would seriously affect the overall findings. There was no obvious link between study quality and outcome. The two most methodologically robust trials Gray et al. [5] and Chien et al. [18] reported different outcomes. The trial that was rated to have the highest risk of bias [19] reported no effect of AT.

We found no significant benefit of AT over usual treatment on adherence attitudes and behaviour. This observation is not consistent with the findings of AT trials that are not included in this review. For example, in a forensic sample, Cavezza et al. [35] found significant effects of AT on both adherence and adherence attitudes in addition to psychiatric symptoms at 3-month follow-up. One of the main reasons for not observing a significant effect on adherence in this review might be the widely recognised difficulty in objectively measuring patients’ treatment adherence [37]. Each of the three reviewed studies that assessed adherence used a different method none of which has been validated as superior to others. Consequently, our finding might simply reflect the questionable ability of adherence scales to objectively measure this behaviour.

It is peculiar that an intervention focused on addressing adherence was found no better than usual care in improving adherence or patients’ attitudes to taking their medication. One reason for this observation might be that the trials were not designed with sufficient power to measure subtle changes in adherence behaviours and attitudes. This review provides outcome information which could be used in future studies as a basis for power calculations allowing identification of improvements in adherence and attitudes. Another factor contributing to our finding might be a ceiling effect due to inclusion of mostly adherent patients in Gray et al. [5] and Schulz et al. [20] and ‘highly motivated’ although non-adherent patients in Chien et al. [18]. Patients in other trials were reported having generally positive attitudes or satisfaction with medication [19, 21] and [26]. As a result, a ceiling effect might have occurred, allowing little room for improvement of adherence in these patients. This observation is consistent with the findings of a review of interventions addressing adherence by Barkhof et al. [38] who suggested that recruiting moderately adherent patients might not provide sufficient potential for change. Conversely, motivated patients might be more likely to improve adherence to treatment after receiving a psychological person-centred intervention such as AT. Adherent and highly motivated patients are not representative of the population of schizophrenia patients [3]. Future trials should focus on recruiting primarily non-adherent patients.

Another interesting finding is that little effects of AT on adherence attitudes and behaviours were observed even in studies that reported significant improvement in negative symptoms and functioning, e.g. Chien et al. [18]. This observation might be explained by the intervention’s mechanism of actions, as targeted minor changes in attitudes or behaviour might potentially have an exponential influence on symptom improvement. Alternatively, AT might have an empowering effect on patients through the use of CBT and MI therapeutic approaches inherent in AT, making patients feel more in control of their illness. Patients’ functioning and symptom control might improve as a result, while adherence itself could actually be less important. Future research should explore the mechanisms of the effect of AT to explain such observations and to ensure that the intervention can be applied in the most appropriate circumstances.

### Review limitations

This review had a few limitations. A relatively small number of randomised controlled studies have been conducted, which used varying lengths of follow-up. Further research is therefore required to allow generalisation of these findings to wider and more diverse populations. The trials included in this review used a variety of different patient-reported measures for similar clinical outcomes and therefore the results of pooled effect sizes should be treated with caution. The exception to this is the measurement of psychiatric symptoms, as the PANSS was used as an outcome measure in five of the six included studies. A further limitation is the underpowered sample size in one of the studies that might increase its risk of Type II error.

## Conclusions

This review provides evidence that adherence therapy can effectively improve patients’ psychiatric symptoms when provided by trained professionals in combination with usual care. It should be noted that although AT did not result in the recommended 20 % reduction in PANSS scores, it can be beneficial to patients when provided in addition to usual antipsychotic treatment. We suggest that patients with schizophrenia would benefit from receiving AT as an adjunct therapy, especially if they have exhibited positive attitudes or moderate adherence to medication. The evidence on the effect of AT on patients’ adherence behaviours and attitudes is limited at best and requires further investigation.

While this brief psychological intervention based on motivational interviewing has a potential to improve patient outcomes, it is unclear whether and how it could be beneficial to non-adherent patients. Robust long-term studies involving representative samples of patients should be conducted with power calculations based on the outcomes of this review, in order to allow exploring the effects of AT on their adherence behaviours and attitudes. Future research should also investigate the therapeutic mechanisms of AT, specifically how the intervention affects patients’ attitudes towards the illness and its treatment and what the relationships are between treatment attitudes, adherence behaviours and patient functioning and symptoms. Improved understanding of these mechanisms could explain why only minor improvements in these areas seemed to result in significant reductions in psychiatric symptoms. Only one trial had a control intervention (health education) that was not TAU. Future research should, therefore, compare AT with an appropriate placebo treatment delivered to non-adherent patients.

### Ethics

Ethical approval and participant consent were not required for this systematic review, since the study involved review and analysis of previously published data.

### Availability of supporting data

The data and materials used in this review are available on request.

## Additional file

Additional file 1: Title: Search strategy details. Description: Provides details of search strategy. (DOCX 16 kb)

## Abbreviations

AT: adherence therapy
BAP: British Association for Psychopharmacology
NICE: National Institute for Health and Care Excellence
PRISMA: preferred reporting items for systematic reviews and meta-analyses
WHO: World Health Organization
