# Supplementary material for: Is adherence therapy an effective adjunct treatment for patients with schizophrenia spectrum disorders? A systematic review and meta-analysis
Source: BMC Psychiatry. 2016 Apr 6;16:90. doi: 10.1186/s12888-016-0801-1 (PMC4822226; doi:10.1186/s12888-016-0801-1)
Supplement: Additional file 1: — Title: Search strategy details. Description: Provides details of search strategy. (DOCX 16 kb) [file 12888_2016_801_MOESM1_ESM.docx]

# Supplementary document: Search strategy details

**Embase (Ovid) search:**

1. adherence therapy.mp. [mp=title, abstract, heading word, drug trade name, original title, device manufacturer, drug manufacturer, device trade name, keyword]

2. schizophrenia/

3. 1 and 2

**Medline (EBSCOhost) search**

S1. (MH “Schizophrenia”)

S2. adherence therapy

S3. S1 AND S2

**PubMed**

"schizophrenia"[MeSH Major Topic] AND "adherence therapy"[All Fields]

**Cochrane Library**

#1 MeSH descriptor: [Schizophrenia] this term only

#2 adherence therapy:kw

#1 and #2

**CINAHL with Full Text**

S1. (MH “Schizophrenia”)

S2. Adherence therapy

S3. S1 AND S2

**Scopus**

#1 TITLE-ABS-KEY(“adherence therapy”)

#2 INDEXTERMS (“schizophrenia”)

#3 #1 AND #2
